# A Demonstration of Selected Skin Cases

**Published:** 1908-03-14

**Authors:** G. Norman Meachen

**Affiliations:** Physician to the Skin Department of the Hospital, and Lecturer in Dermatology at the North-East London Post-Graduate College.


### A DEMONSTRATION OF SELECTED SKIN CASES.

By G. NORMAN MEACHEN, M.D., B.S., M.R.C.P., Physician to the Skin Department of the Hospital, and Lecturer in Dermatology at the North-East London Post-Graduate College.

(Given at the Prince of Wales's General Hospital, Tottenham.)

Ladies and Gentlemen,—This boy, aged 11, is one of two brothers who are affected with patchy baldness of several months' duration. The areas are irregular in outline, and their bases are moderately smooth, though they have not quite the billiard-ball like polish usually associated with alopecia areata. I have carefully examined the scalp for evidences of ringworm, because this particular variety of alopecia is simulated by tinea decalvans, or "bald" ringworm. There are no spores present, but on close inspection of the patches you will notice several short hairs which are not broken off short and bent irregularly, as are ringworm hairs, but are thickened at their extremities, and under the microscope you can see that the end is frayed out and brushlike. Quite by accident I discovered that one of the hairs was distinctly nodose, or moniliform, and this affection of the hair-shaft is very rare. Of the two rival theories as to the causation of alopecia areata, the parasitic and the neuro-trophic, the latter would appear to be more supported by facts. Inoculation experiments in man have not been successful, in spite of the fact that the micro-bacillus of seborrhœa is frequently found in association with alopecia, whereas there are numerous instances on record, some of which I have noted personally, where there have been evidences of peripheral nerve-irritation affecting some branch of the fifth cranial nerve, such as severe dental caries, errors of refraction, or neuralgia. When one or other of these sources of irritation has been removed, it would seem as though an impetus has been given thereby to the growth of hair. At the same time, one cannot dispense with local applications, and the one I am using for this boy is oil of cinnamon, in the strength of two drachms to the ounce of olive oil. Oil of cassia, a cheaper substitute, is not nearly so efficacious.

### Some More Scalp Cases.

Here is a little girl, aged 4, who has a generalised thinning of the hair after an acute illness, in this case whooping-cough. Her hair is very thin, and what there is does not seem very well nourished. In this variety of alopecia coming on after a severe or debilitating malady I think chloral hydrate is a valuable stimulant locally applied. She is having an ointment containing 10 grains of the drug and 2 drachms of the B.P. red oxide of mercury ointment, in 1 ounce of lanoline. It is often necessary to give a tonic as well, such as the syrup of the phosphate of iron.

This other child, aged 4, had a condition of fine scaliness of almost the entire scalp when I saw her in my clinic last week. Such an affection is very commonly diagnosed as ringworm, but no spores have been found in this case. Moreover, there are a few small crusts here and there, and she has some blepharitis in addition. It is simply a case of scalp eczema. The converse mistake, that of calling a ringworm eczema, is, of course, frequently made, for the characteristic stumps are not often seen when there is much general scaliness. In cases of doubt it is advisable to take a surface-scraping of the scalp and examine it in potash under the microscope. I have prescribed a weak ointment of ammoniated mercury.

### Satin-wood Dermatitis.

This man, aged 43, is a cabinet-maker, and he has been engaged in the work for upwards of twelve years. It is only during the last three months that he has been working in satin-wood, and he has suffered for the last month with a severe eczematous dermatitis affecting the hands, neck, and face. The satin-wood is used for inlaid work, being sandwiched between other softer kinds of wood, and part of this man's work has been to plane it down evenly, and it is the fine dust which rises during this process which is prone to irritate those with sensitive skins. A few cases have been recorded from time to time of this particular "trade dermatitis," and the only thing which seems to have any permanently good effect is complete removal of the source of irritation. He has brought us a few samples of the wood, and also of other woods inlaid with pewter. Working with rubber gloves may be permitted, but a short abstinence from work is imperative in acute cases. He has found relief from bathing with a lotion of calamine and cyllin (1 per cent.), equal parts, after which an ointment containing 15 grains of ichthyol in Lassar's paste has been kept constantly applied to the affected parts.

### Urticaria Papulosa with Hæmophilia.

I am showing you this little girl, aged 2, for two reasons. In the first place, she is a typical example of a very common skin eruption in children which the older dermatologists described as "Lichen urticatus." The primary lesion is a small papular wheal, found upon the lumbar and sacral regions, but in bad cases nearly the whole body and limbs may be affected. Irritation is great, so that the papule quickly becomes surmounted by a minute blood-crust, the result of scratching. When the lesions are undergoing involution they get flattened, and then assume a distinctly lichenoid aspect. Some of these you can now see upon the back, and in the absence of any primary lesion one can understand, perhaps, that the condition might pass for a lichen planus, but this affection is very rare in infants. Secondly, we are informed by the mother that the child is a "bleeder," and that she lost one other child with hæmophilia. The present child has only been in England eight weeks, having been an out-patient at an American hospital. She has suffered from severe epistaxis, but the interesting point is that profuse hæmorrhage is said to have occurred as a result of scratching the skin lesions. I cannot recall any instance in which fatal hæmorrhage has taken place in a hæmophilic solely as the result of scratching, but the possibility causes one to take a serious view of the situation. I have given her a mixture containing 5 grains of calcium chloride, the taste of which is disguised by a little extract of liquorice and syrup of orange. Locally a dilute cyllin lotion or bath is very beneficial, after which an ointment containing a little ichthyol or beta-naphthol may be applied to the spots.

### Tertiary Syphilitic Ulceration.

This man, a gardener, aged 39, has a peculiar ulceration of the scrotum and adjacent part of the perinæum. When I first saw him three weeks ago the integument over the lower part of the penile urethra, the scrotum and perinæum was deeply ulcerated, but there were no lesions elsewhere, except a shallow ulcer on the under surface of the tongue. The primary infection occurred in Cape Town nine years ago, and he was treated for only three months. He attended here eighteen months ago with a few pustular lesions upon the legs. I have given him iodipin (10 per cent.) in drachm doses three times a day in an emulsion, and I really think that this remedy has produced a more favourable impression in the short time that he has had it than would have been the case with potassium iodide. At any rate, the drug is a most useful one in causing rapid absorption of tertiary specific lesions, and it has the marked advantage of producing no symptoms of iodism.

### Extensive Tinea Versicolor.

It is sometimes said that the value of colour in the diagnosis of a cutaneous eruption has been greatly over-estimated, and with that statement I am inclined to agree. Nevertheless, the peculiar dirty, yellowish-brown tint of this man's rash is, in itself, a sufficiently diagnostic feature of tinea or pityriasis versicolor. You will notice that the rash is very extensive, and that upon the back the individual circinate lesions have run together to form one large sheet. We found the microsporon furfur in abundance upon taking a surface-scraping. There has been practically no irritation at all in this case, and it is quite possible for a patient to have the disease extensively and to be unaware of the fact. The history of medicine is always interesting, and I will read you a few words from Dr. Willan's text-book, published in 1824—*i.e.*, twenty-two years prior to the discovery of the fungus by Eichstedt. "The causes of this pityriasis," he writes, "are not well understood. It occurs most frequently in those who have resided in hot climates, especially in its troublesome form. The most extensive eruption that I have seen occurred in a Custom-house officer, after drinking spirits freely during a day of fasting in the boat on the Thames. Fruit, mushrooms, sudden alternations of heat and cold, violent exercise with flannel next to the skin, have been mentioned as probable causes of this eruption." This allusion to flannel reminds me that the so-called "flannel-rash" may be due to this disease, but more often it is a seborrhœic dermatitis. Other authors of the period confused tinea versicolor with chloasma, or pigmentation of the skin. The fungus is readily found on examination, and is, of course, proof positive of the nature of the eruption. The disease is easily cured by frequently bathing the affected parts with warm solutions of thiosulphate of soda (ordinary photographers' "hypo") in the strength of a drachm or two to the ounce of water.

### Some Cases of Psoriasis.

I have several cases of psoriasis here this afternoon which illustrate certain points in connection with that disorder.

This man, aged 42, says that the rash first appeared upon and in the neighbourhood of the scar of an old compound fracture of the leg, and now you may see the scaly patches actually upon the scar. It is not so very uncommon for psoriasis to appear upon scar-tissue first of all and then to spread to other parts.

This woman, aged 35, has had the disease for three years, generally worse in the autumn, and she also has a rheumatic history. Such a case is nearly always benefited by salicin or drugs of that group, and she has been taking acetosalicylic acid in 10-grain doses three times a day for some time.

Here is a case in a younger patient, and her scalp is markedly affected. The lesions resemble splashes of mortar, and scalp-psoriasis is strikingly different from seborrhœa of this part, the scaliness in which is far more diffuse, less silvery, and more greasy. This girl is not well, suffering much from dyspepsia and constipation. That psoriasis cannot now be regarded as the "disease of the healthy," as was originally propounded by Hebra, is beginning to be more and more recognised, and my friend Dr. Abraham has shown that in a considerable percentage of psoriatics there is some systemic disorder concomitant with the skin eruption. It behoves us, then, to examine the other systems of the body very carefully in this disease, as in any widespread cutaneous affection. The local treatment of all these patients has been bathing with cyllin (creolin) and the inunction of ointments containing the same drug with a little salicylic acid.

### Dysidrosis Palmarum et Plantarum.

This boy, aged 11, has suffered for the last six weeks with a troublesome form of sweat-eruption of the palms and soles. The condition began about the toes; the skin in between speedily became sodden and eczematous, rendering walking almost impossible, and some bromidrosis ultimately developed. Then the hands became affected. He is now very much better, the feet being practically well. I gave him baths of cyllin for the feet, and after gently drying the toes, the compound boracic powder was thickly dusted on. At his next visit he was given an ointment containing a little iodoform for application to the toes, while the hands were directed to be kept covered with a mild Lassar's paste.

